# Characterization of a *Cul9–Parkin* double knockout mouse model for Parkinson’s disease

**DOI:** 10.1038/s41598-020-73854-y

**Published:** 2020-10-09

**Authors:** Emilie Hollville, Valerie Joers, Ayumi Nakamura, Vijay Swahari, Malú G. Tansey, Sheryl S. Moy, Mohanish Deshmukh

**Affiliations:** 1 Neuroscience Center, University of North Carolina, Chapel Hill, NC USA; 2 Department of Neuroscience, Center for Translational Research in Neurodegenerative Disease, University of Florida, Gainesville, FL USA; 3 Department of Psychiatry, University of North Carolina, Chapel Hill, NC USA; 4 Carolina Institute for Developmental Disabilities, University of North Carolina, Chapel Hill, NC USA; 5 Department of Cell Biology and Physiology, University of North Carolina, Chapel Hill, NC USA

**Keywords:** Neuroscience, Diseases of the nervous system, Parkinson's disease

## Abstract

Mitochondrial quality control is essential for the long-term survival of postmitotic neurons. The E3 ubiquitin ligase Parkin promotes the degradation of damaged mitochondria via mitophagy and mutations in Parkin are a major cause of early-onset Parkinson’s disease (PD). Surprisingly however, mice deleted for *Parkin* alone are rather asymptomatic for PD-related pathology, suggesting that other complementary or redundant mitochondrial quality control pathways may exist in neurons. Mitochondrial damage is often accompanied by the release of toxic proteins such as cytochrome *c*. We have reported that once in the cytosol, cytochrome *c* is targeted for degradation by the E3 ligase CUL9 in neurons. Here we examined whether CUL9 and Parkin cooperate to promote optimal neuronal survival in vivo. We generated mice deficient for both *Cul9* and *Parkin* and examined them for PD-related phenotypes. Specifically, we conducted assays to examine behavioural deficits (locomotor, sensory, memory and learning) and loss of dopaminergic neurons in both males and females. Our results show that the loss of *Cul9* and *Parkin* together did not enhance the effect of *Parkin* deficiency alone. These results indicate that while both Parkin and CUL9 participate in mitochondrial quality control, neurons likely have multiple redundant mechanisms to ensure their long-term survival.

## Introduction

Parkinson’s disease (PD) is a neurodegenerative disorder primarily characterized by motor dysfunction. Early loss of dopaminergic neurons in the substantia nigra pars compacta (SNpc) of PD patients leads to a deficiency in dopamine that causes slow and progressive resting tremor, slowness of movement (bradykinesia), muscular rigidity, postural instability and restless movements (akathisia)^1,2^. Importantly, non-motor symptoms are also observed. These include gastrointestinal, sleep and olfactory dysfunction, cognitive impairment, depression and apathy, some of which develop before the emergence of classical motor symptoms^1,3–5^. The vast majority (90%) of PD cases are sporadic and are potentially associated with environmental risk factors including exposure to pesticides and non-pesticide toxicants (heavy metals and solvents), methamphetamine use and traumatic brain injury^6,7^. In addition, familial or monogenic forms of the disease due to mutations in high risk genes are seen in 5–10% of PD patients and are mainly associated with an early onset form of the disorder^1,2^.

Mutations in *PARK2*, which encodes the E3 ubiquitin ligase Parkin, are the most common cause of early onset autosomal recessive PD. Parkin, in concert with the serine/threonine kinase PINK1 (*PARK6*), participates in a mitochondrial quality control pathway, whereby damaged mitochondria are eliminated by macro-autophagy in a process called mitophagy^8–14^. Parkin is a promiscuous ubiquitin ligase capable of ubiquitinating multiple mitochondrial proteins. Recognized by ubiquitin-binding proteins (e.g. NDP52, optineurin, p62/SQSTR2), the ubiquitinated proteins on the surface of damaged organelles are tethered to forming autophagosomes^15–18^. Following extension of the autophagosomal membranes, the autophagosome fuses with a lysosome to promote the clearance of the engulfed mitochondria.

Multiple elements point toward mitochondrial dysfunction in PD. Early reports showed that the neurotoxin MPP+, a derivative metabolite of MPTP that targets the mitochondrial NADH dehydrogenase, specifically induces the death of dopaminergic neurons in the SNpc^19,20^. In addition, deficits in mitochondrial complex I are typically found in post-mortem brains of PD patients^21,22^. Consistently, the lack of *Parkin* results in mitochondrial dysfunction including reduced respiration and decreased ATP production in rodent brains and in in vitro cultures from *Parkin* mutant mice^23–25^. Surprisingly, however, mice deficient in *Parkin* alone do not develop a strong motor dysfunction phenotype, nor do they display accelerated dopaminergic neuron loss^26–29^. Thus, mice deficient in *Parkin* alone are not considered a robust model of PD^30^.

Reduction of oxidative phosphorylation activity can result in the abnormal production of reactive oxygen species and loss of mitochondrial membrane potential. More importantly, dysfunctional mitochondria can release harmful mitochondrial proteins such as cytochrome* c* (cyt *c*) in the cytosol. Indeed, the release of mitochondrial cyt* c* has been observed in MPTP mouse models of PD^31,32^. The presence of cyt* c* in the cytosol is particularly toxic to neurons because it can trigger caspase activation and apoptotic cell death^33^. Cytosolic cyt* c* can additionally impair calcium homeostasis through the binding to IP3R on the endoplasmic reticulum, ultimately resulting in synaptic dysfunction and neuronal degeneration^34–36^. Importantly, cytoplasmic cyt* c* can also trigger the oligomerization of α-synuclein (*PARK1*)^37^. This is particularly relevant to PD as aggregation of α-synuclein into Lewy bodies is a major pathological hallmark of the disease^1,2^.

We have previously shown that mitochondrially-released cyt* c* can be targeted for degradation via the proteasome. In neurons, the degradation of cytosolic cyt* c* is mediated by the ubiquitin ligase cullin-9 (CUL9)^38^. CUL9 belongs to the Cullin family, the members of which form multimodular complexes that are involved in the ubiquitination of multiple substrates associated with a wide range of cellular functions^38–40^. The degradation of cytosolic cyt* c* by CUL9 is important for neuronal survival, as neurons deficient in *Cul9* are more sensitive to mitochondrial damage^38^.

Mechanisms such as Parkin-mediated mitophagy and CUL9-mediated cyt* c* degradation can be particularly important for long-term survival of postmitotic neurons, which are likely to experience mitochondrial stress throughout the organism’s life^41^. Optimal mitochondria quality control could involve both the removal of the damaged mitochondria *as well as* the degradation of any mitochondrial content that may have leaked out of the damaged mitochondria. Thus, despite the accumulation of dysfunctional mitochondria, the lack of an overt neuronal phenotype due to the deletion of *Parkin* alone could be due to the presence of complementary mitochondrial quality control pathways. Here we examined whether CUL9 together with Parkin could function in concert to protect neurons against mitochondrial insults. To this end, we generated *Cul9* and *Parkin* double knockout (KO) mice and analysed the consequences of deleting both E3 ligases by examining early PD-related behaviours.

## Results

### CUL9 remains cytosolic in response to mitochondrial depolarization

**Figure 1:**
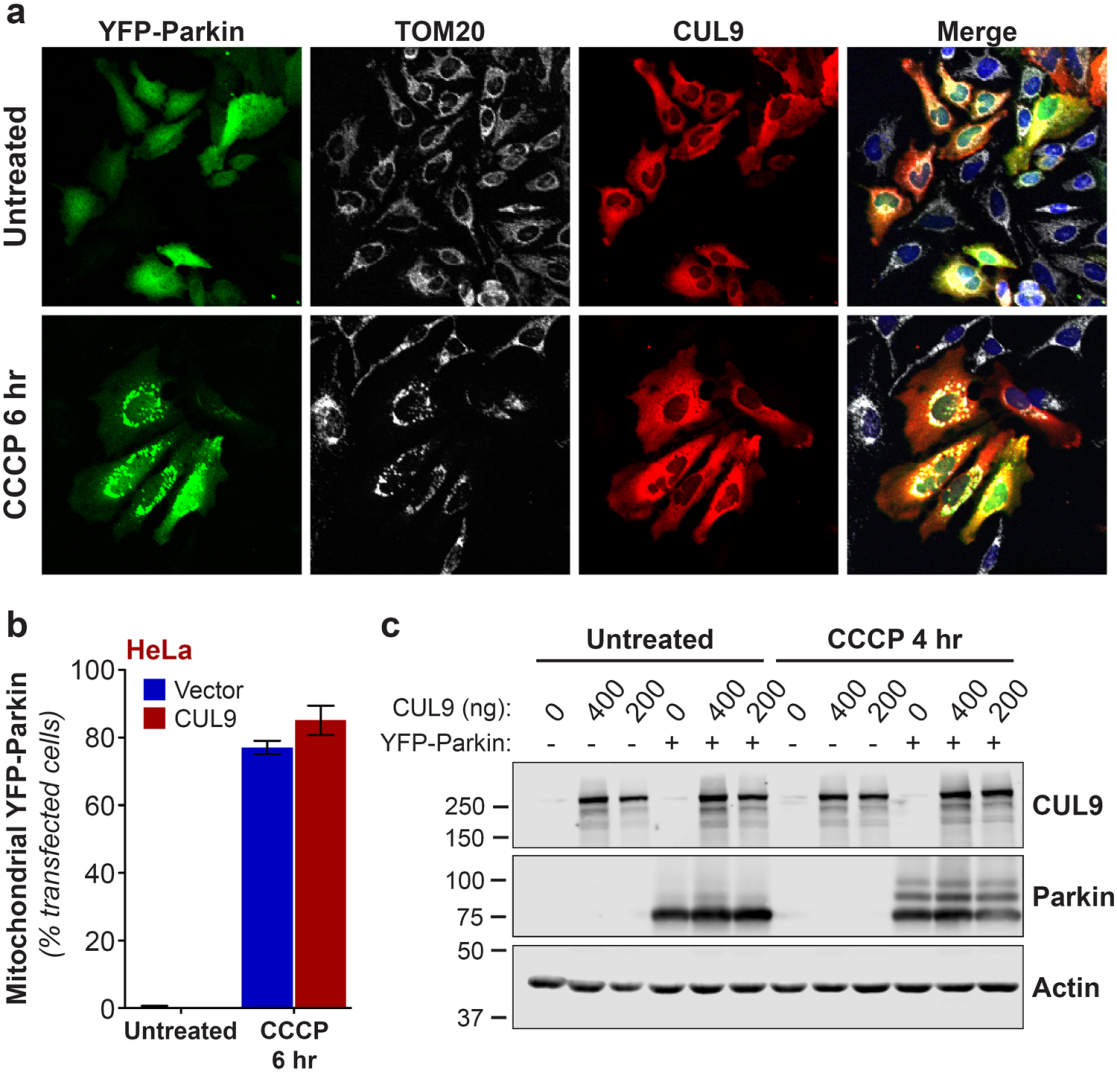
CUL9 remains cytosolic upon mitochondrial depolarization. HeLa cells were transfected with YFP-Parkin cDNA (200 ng) with or without CUL9 cDNA (400 ng) and treated with CCCP (10 µM) for 6 h. (**a**) Cells were immunostained for mitochondria (TOM20, grey) and CUL9 (red) and (**b**) co-localization between Parkin and mitochondria was scored among YFP-Parkin-positive cells by confocal microscopy. Counts from 3 independent fields of view in a representative experiment (*n* = 3) are shown. Mean ± SEM are shown. (**c**) HeLa cells were transfected with the indicated amount of plasmid encoding CUL9 along with YFP-Parkin construct (200 ng). Cells were treated with CCCP (10 µM) for 4 h and whole-cell lysates were analysed by immunoblotting. A representative immunoblot from 3 independent experiments is shown. Full-length immunoblots are presented in Supplementary Fig. S1.

Our previous results indicated that CUL9 can degrade cyt* c* that is released from damaged mitochondria^38^, suggesting that it could play a role in mitochondrial quality control. As several E3 ubiquitin ligases known to be involved in mitochondrial homeostasis (e.g. Parkin, Huwe1/Mule and XIAP) are recruited to depolarized mitochondria^42,43^, we tested whether CUL9 also translocated to uncoupled mitochondria. We also examined whether CUL9 and Parkin affected the translocation of one another to uncoupled mitochondria. To this end, we expressed CUL9 in the presence or absence of Parkin and induced mitochondrial depolarization with the protonophore CCCP (carbonyl cyanide m-chlorophenylhydrazone). As anticipated, exogenously expressed Parkin is rapidly recruited to mitochondria (Fig. 1a,b) and ubiquitinated (Fig. 1c) upon CCCP treatment. In contrast, CUL9, which is localized in the cytosol of resting cells, did not translocate to mitochondria in response to CCCP-induced uncoupling (Fig. 1a). In addition, CUL9 did not affect Parkin translocation to mitochondria and activation after CCCP treatment (Fig. 1a–c).

### Generation of *Cul9*, *Parkin* double knockout mice

**Figure 2:**
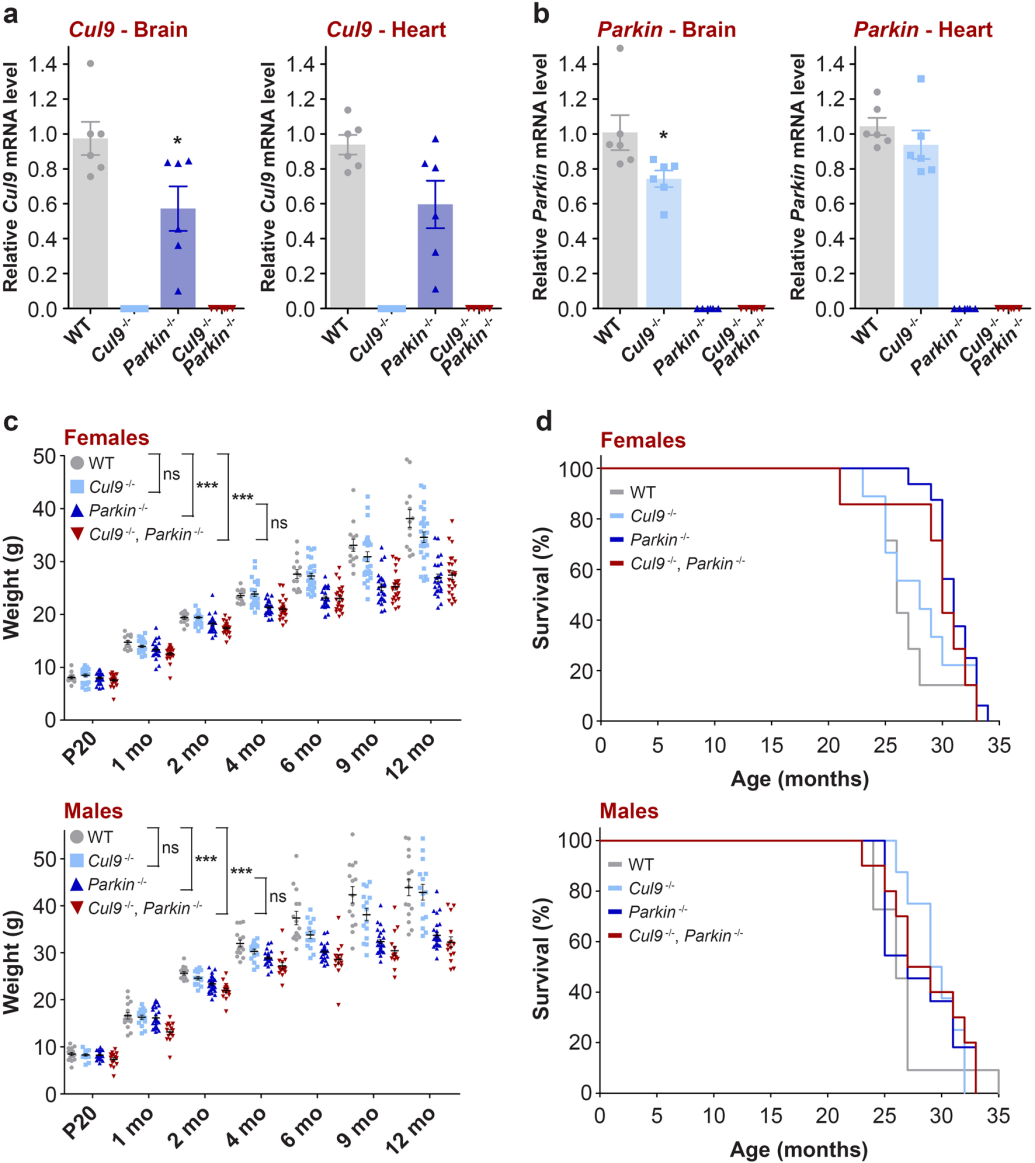
Generation of *Cul9*^*−/−*^, *Parkin*^*−/−*^ double knockout mice. (**a**,**b**) Quantitative analysis of *Cul9* (**a**) and *Parkin* (**b**) mRNA expression by RT-qPCR from brain and heart tissues of *Cul9* and *Parkin* single and double knockout mice (*n* = 6, 3 males and 3 females). Data were normalized to *Gapdh*. Means ± SEM are shown. Unpaired t-test (two-tailed) with Welch's correction (*Cul9* brain: WT vs. *Parkin*^*−/−*^, *p = 0.0321; *Parkin* brain: WT vs. *Cul9*^*−/−*^, *p = 0.0471). (**c**) Measure of *Cul9* and *Parkin* single and double knockout mice weight from postnatal day 20 (P20) to 12 months (females: WT *n* = 13; *Cul9*^*−/−*^* n* = 26; *Parkin*^*−/−*^* n* = 24; *Cul9*^*−/−*^, *Parkin*^*−/−*^* n* = 24), (males: WT *n* = 15; *Cul9*^*−/−*^* n* = 15; *Parkin*^*−/−*^* n* = 28; *Cul9*^*−/−*^, *Parkin*^*−/−*^* n* = 15). Means ± SEM are shown, two-way repeated measures ANOVA with post-hoc Tukey's multiple comparisons test (females: WT vs. *Parkin*^*−/−*^, ***p < 0.0001; WT vs. *Cul9*^*−/−*^, *Parkin*^*−/−*^, ***p < 0.0001), (males: WT vs. *Parkin*^*−/−*^, ***p < 0.0001; WT vs. *Cul9*^*−/−*^, *Parkin*^*−/−*^, ***p < 0.0001), *ns*, non-significant. (**d**) Survival curve of *Cul9* and *Parkin* single and double knockout mice (females: WT *n* = 10; *Cul9*^*−/−*^* n* = 9; *Parkin*^*−/−*^* n* = 16; *Cul9*^*−/−*^, *Parkin*^*−/−*^* n* = 7), (males: WT *n* = 11; *Cul9*^*−/−*^* n* = 8; *Parkin*^*−/−*^* n* = 11; *Cul9*^*−/−*^, *Parkin*^*−/−*^* n* = 10). *n* represents the number of independent animals.

To test the hypothesis that both CUL9 and Parkin could cooperate to maintain mitochondrial homeostasis, we crossed *Cul9*^−/−^ with *Parkin*^−/−^ mice to obtain *Cul9*, *Parkin* double knockout mice (*Cul9*^−/−^, *Parkin*^−/−^). As expected, the expression of both *Cul9* and *Parkin* was lost in these animals (Fig. 2a,b). Analysis of levels of *Cul9* and *Parkin* mRNA in brain tissue of *Parkin*^−/−^ and *Cul9*^−/−^ mice respectively, shows a reduction in *Cul9* expression in *Parkin*^−/−^ brains, which could indicate a potential genetic interaction between both enzymes (Fig. 2a).

Similar to mice deleted for *Cul9* and *Parkin* alone, the *Cul9*, *Parkin* double knockout mice were born at the expected Mendelian ratio, were viable and fertile. Their development appeared normal, although the body weight of *Cul9*^−/−^, *Parkin*^−/−^ mice was significantly reduced compared to wild-type animals, especially after 6 months of age, as previously reported for the *Parkin*^−/−^ mice^23,28^ (females: WT *n* = 13; *Cul9*^−*/*−^* n* = 26; *Parkin*^−*/*−^* n* = 24; *Cul9*^−*/*−^, *Parkin*^−*/*−^* n* = 24; WT vs. *Parkin*^−*/*−^, ***p < 0.0001; WT versus *Cul9*^*−/−*^, *Parkin*^*−/−*^, ***p < 0.0001), (males: WT *n* = 15; *Cul9*^*−/−*^* n* = 15; *Parkin*^*−/−*^* n* = 28; *Cul9*^*−/−*^, *Parkin*^*−/−*^* n* = 15; WT vs. *Parkin*^*−/−*^, ***p < 0.0001; WT vs. *Cul9*^*−/−*^, *Parkin*^*−/−*^, ***p < 0.0001) (Fig. 2c). Kaplan–Meier survival analysis of these animals did not reveal any reduced life span compared to wild-type or to single knockout controls (females: WT *n* = 10; *Cul9*^*−/−*^* n* = 9; *Parkin*^*−/−*^* n* = 16; *Cul9*^*−/−*^, *Parkin*^*−/−*^* n* = 7; males: WT *n* = 11; *Cul9*^*−/−*^* n* = 8; *Parkin*^*−/−*^* n* = 11; *Cul9*^*−/−*^, *Parkin*^*−/−*^* n* = 10) (Fig. 2d).

### Behavioural analyses of the *Cul9*, *Parkin* double knockout mice

To determine whether *Cul9* deficiency can enhance *Parkin* loss of function, we generated a cohort of 10 male and 10 female mice deficient in *Cul9* or *Parkin* alone or mice deficient in both *Cul9* and *Parkin*. These cohorts, along with wild-type mice, were tested for Parkinsonian behavioural deficits in young adults in which *Parkin* deficiency is typically asymptomatic. We focused the behavioural analysis on locomotor, sensory, as well as memory and learning skills, as deficits in these have been seen in PD.

#### Locomotor function in *Cul9*^−/−^, *Parkin*^−/−^ mice

**Figure 3:**
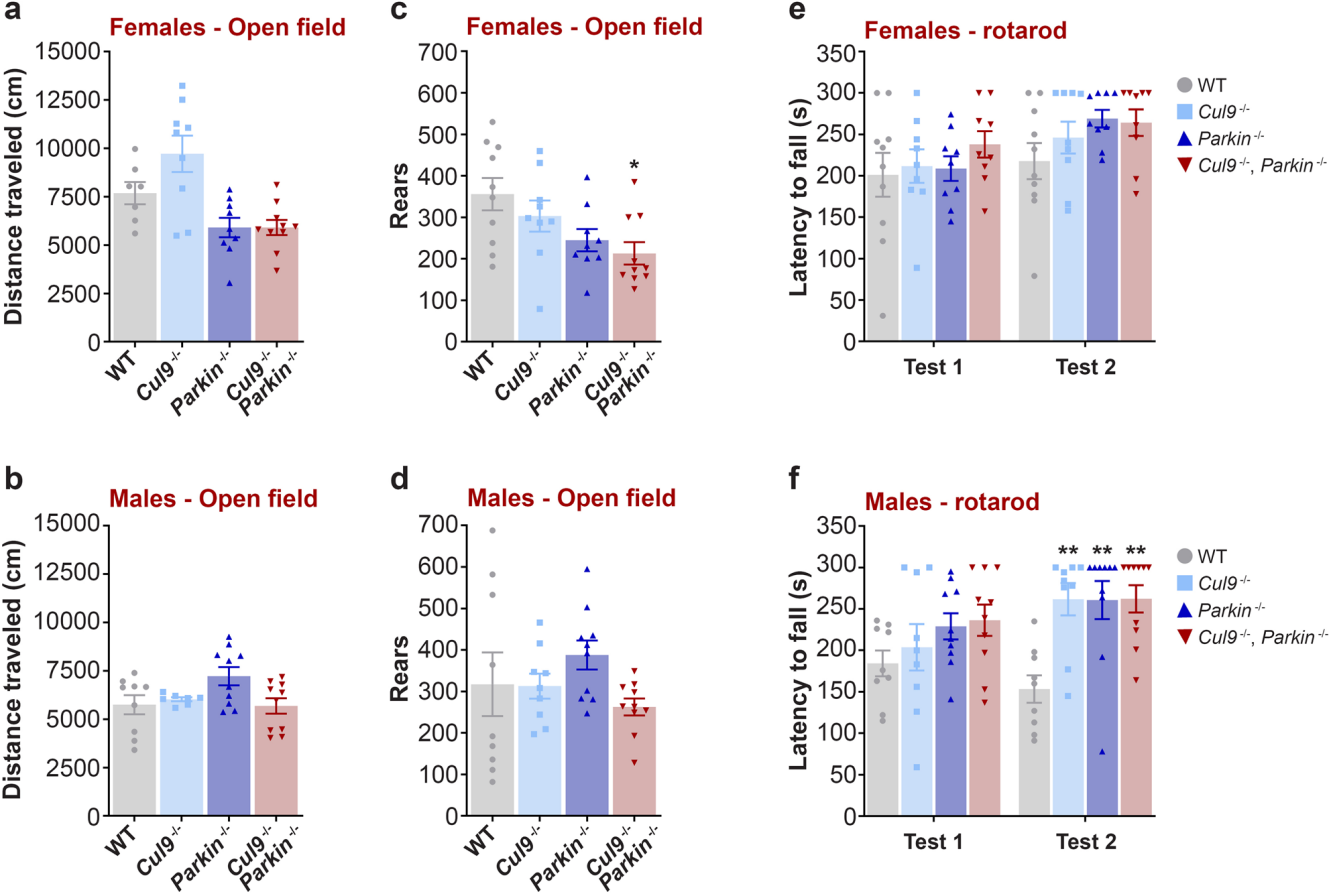
Locomotor function characterization of *Cul9*^*−/−*^, *Parkin*^*−/−*^ mice. (**a**,**b**) Total distance travelled and (**c**,**d**) total number of rears in the open field (1 h) of *Cul9* and *Parkin* single and double knockout mice (3–4 months old), (females distance: *n* = 7–10; males distance: *n* = 7–10; females rears: *n* = 9–10; males rears: *n* = 9–10). One-way ANOVA, with post-hoc Tukey's multiple comparisons test (females rears: WT vs. *Cul9*^*−/−*^, *Parkin*^*−/−*^, *p = 0.0186). (**e**,**f**) Latency to fall from an accelerating rotarod (5 min tests, 48 h between test 1 and test 2) of *Cul9* and *Parkin* single and double knockout mice (4–5 months old), (females: *n* = 9–10; males: *n* = 9–10). Two-way repeated measures ANOVA with post-hoc Tukey's multiple comparisons test (males test 2: WT vs. *Cul9*^*−/−*^, **p = 0.0017; WT vs. *Parkin*^*−/−*^, **p = 0.0014; WT vs. *Cul9*^*−/−*^, *Parkin*^*−/−*^, **p = 0.0011). Means ± SEM are shown, *n* represents the number of independent animals.

General exploratory activity was evaluated in an open field chamber over a sixty minute trial, where the total distance travelled during the test was recorded (females: WT *n* = 7; *Cul9*^*−/−*^* n* = 9; *Parkin*^*−/−*^* n* = 9; *Cul9*^*−/−*^, *Parkin*^*−/−*^* n* = 10; males: WT *n* = 9; *Cul9*^*−/−*^* n* = 7; *Parkin*^*−/−*^* n* = 10; *Cul9*^*−/−*^, *Parkin*^*−/−*^* n* = 10) (Fig. 3a,b, Supplementary Table S1). We found that female, but not male, *Parkin*^−/−^ mice had reduced locomotor activity on average compared to wild-type mice; the difference observed, however, did not reach significance (females WT vs. *Parkin*^*−/−*^, *p* = 0.2591). The locomotor activity of *Cul9*^−/−^, *Parkin*^−/−^ mice was indistinguishable from that of the *Parkin*^−/−^ mice for both sexes.

Similar results were obtained upon analysis of spontaneous vertical activity (rearing) (females: WT *n* = 10; *Cul9*^*−/−*^* n* = 9; *Parkin*^*−/−*^* n* = 9; *Cul9*^*−/−*^, *Parkin*^*−/−*^* n* = 10; males: WT *n* = 9; *Cul9*^*−/−*^* n* = 9; *Parkin*^*−/−*^* n* = 10; *Cul9*^*−/−*^, *Parkin*^*−/−*^* n* = 10) (Fig. 3c,d, Supplementary Table S1). *Parkin*-deficient females, but not males, exhibited a negligible reduction in the number of rears compared to wild-type animals (females WT vs. *Parkin*^*−/−*^, *p* = 0.1046). Rearing, however, was comparable between *Cul9*^−/−^, *Parkin*^*−/−*^ mice and *Parkin*^−/−^ mice in both the male and female groups.

To determine whether the absence of motor deficit was due to the young age of the animals, the same cohort was re-analysed at an older age. However, even when re-tested at 13–14 months of age in the open field for horizontal (females: WT *n* = 8; *Cul9*^*−/−*^* n* = 9; *Parkin*^*−/−*^* n* = 8; *Cul9*^*−/−*^, *Parkin*^*−/−*^* n* = 10; males: WT *n* = 9; *Cul9*^*−/−*^* n* = 7; *Parkin*^*−/−*^* n* = 10; *Cul9*^*−/−*^, *Parkin*^*−/−*^* n* = 10) and vertical activity (females: WT *n* = 10; *Cul9*^*−/−*^* n* = 9; *Parkin*^*−/−*^* n* = 9; *Cul9*^*−/−*^, *Parkin*^*−/−*^* n* = 10; males: WT *n* = 9; *Cul9*^*−/−*^* n* = 7; *Parkin*^*−/−*^* n* = 10; *Cul9*^*−/−*^, *Parkin*^*−/−*^* n* = 9), male and female *Parkin* KOs did not show reduced locomotor function (Supplementary Fig. S2a–d, Supplementary Table S1). Also, the *Cul9*, *Parkin* double KO were not markedly different when compared to the *Parkin* KO mice in both sexes (Supplementary Fig. S2a–d).

To further characterize the locomotor function of the young adult mice, we tested their motor coordination and balance by measuring the latency to fall from a rotating rod (females: WT *n* = 10; *Cul9*^*−/−*^* n* = 9; *Parkin*^*−/−*^* n* = 9; *Cul9*^*−/−*^, *Parkin*^*−/−*^* n* = 9; males: WT *n* = 9; *Cul9*^*−/−*^* n* = 9; *Parkin*^*−/−*^* n* = 10; *Cul9*^*−/−*^, *Parkin*^*−/−*^* n* = 10) (Fig. 3e,f, Supplementary Table S1). Rather surprisingly, while there was no significant difference in performance between mutant and wild-type females, all mutant males exhibited increased motor coordination compared to wild-type males (males WT vs. *Cul9*^*−/−*^, **p = 0.0017; WT vs. *Parkin*^*−/−*^, **p = 0.0014; WT vs. *Cul9*^*−/−*^, *Parkin*^*−/−*^, **p = 0.0011). However, *Cul9*^−/−^, *Parkin*^*−/−*^ and *Parkin*^−/−^ males were indistinguishable.

Tested again at 11–12 months of age, mice performed similarly in the rotarod test (females: WT *n* = 10; *Cul9*^*−/−*^* n* = 9; *Parkin*^*−/−*^* n* = 9; *Cul9*^*−/−*^, *Parkin*^*−/−*^* n* = 10; males: WT *n* = 9; *Cul9*^*−/−*^* n* = 8; *Parkin*^*−/−*^* n* = 10; *Cul9*^*−/−*^, *Parkin*^*−/−*^* n* = 10) (Supplementary Fig. S2e, S2f, Supplementary Table S1). The females of all mutant groups were comparable to the control female group, while the mutant males showed a partial increase in motor coordination performance (males WT vs. *Cul9*^*−/−*^, *Parkin*^*−/−*^, *p = 0.0173). However, no difference between *Cul9*^−/−^, *Parkin*^*−/−*^ mice and *Parkin*^−/−^ males was detected even at this older age.

Additionally, a wire-hang test for grip strength, performed at 3–4 months of age, revealed no defect across all groups: all of the mice were able to remain suspended from a cage lid for the maximum trial time (data not shown). Together, these results indicate that the absence of *Cul9* does not aggravate the locomotor function of *Parkin*-deficient mice.

#### Sensory function in *Cul9*^−/−^, *Parkin*^−/−^ mice

Non-motor symptoms often precede the onset of motor deterioration in PD patients. Among these symptoms, alteration of multiple sensory functions has been widely reported, including heat/cold intolerance, increased pain, and loss of taste and smell^4,44^. We therefore examined whether non-motor functions could be affected in the adult *Cul9*, *Parkin* double mutant mice.

**Figure 4:**
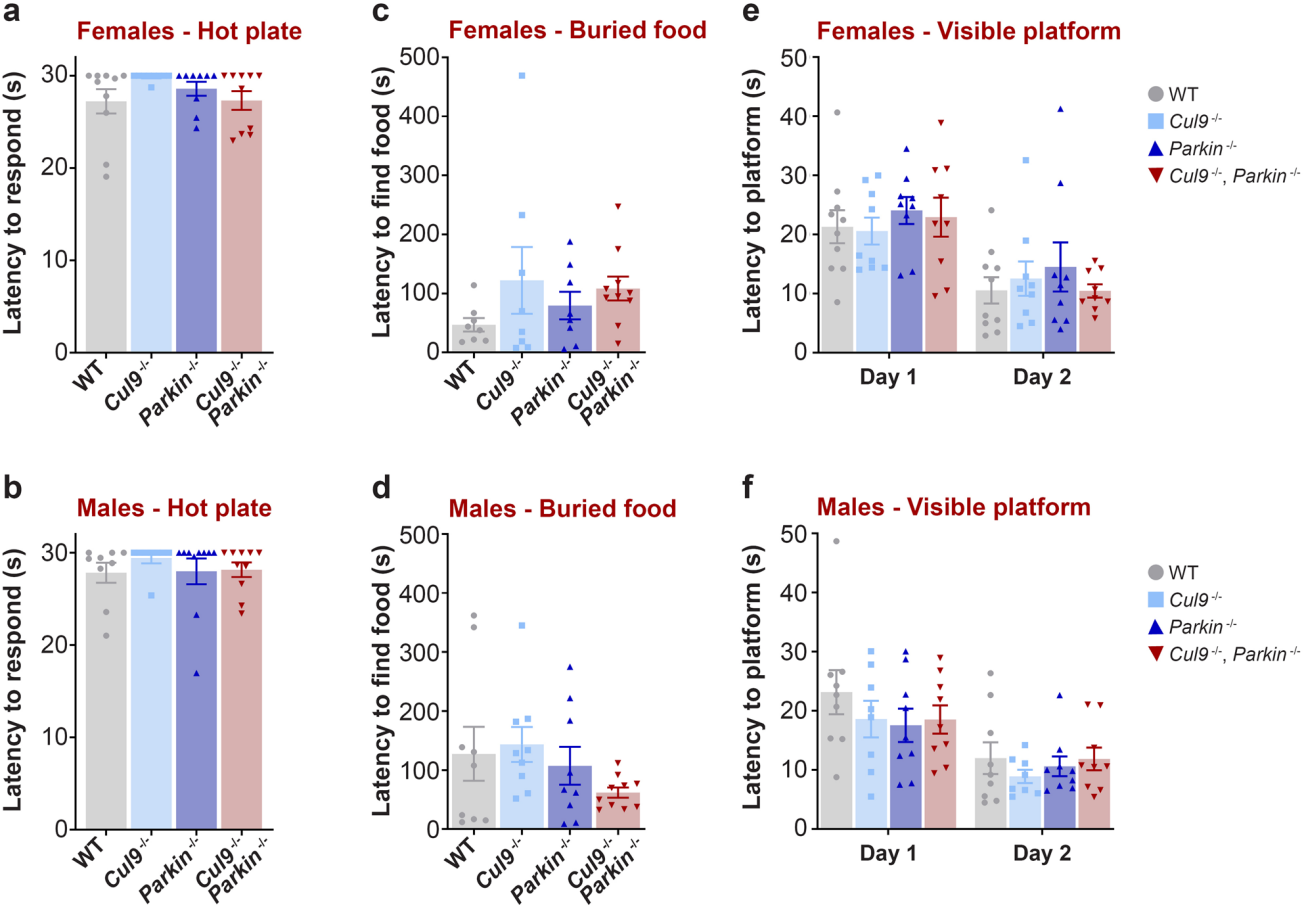
Sensorimotor function characterization of *Cul9*^*−/−*^, *Parkin*^*−/−*^ mice. (**a**,**b**) Response latency after exposure to a hot surface (55 °C, 30 s maximum trial) of *Cul9* and *Parkin* single and double knockout mice (6–7 months old), (females: *n* = 9–10; males: *n* = 8–10). (**c**,**d**) Latency to find buried food (15 min trial) of *Cul9* and *Parkin* single and double knockout mice (4–5 months old), (females: *n* = 8–10; males: *n* = 9–10). (**e**,**f**) Latency to find a visible platform in a Morris water maze (60 s trials; 4 trials per day) of *Cul9* and *Parkin* single and double knockout mice (4–5 months old), (females: *n* = 9–10; males: *n* = 8–9). Means ± SEM are shown, *n* represents the number of independent animals.

To test mice for thermal nociception, the cohort was subjected to a hot plate test. The animals were exposed to a plate heated at 55 °C and the latency to react to the hot plate was measured, with a maximum test length of thirty seconds (females: WT *n* = 10; *Cul9*^*−/−*^* n* = 9; *Parkin*^*−/−*^* n* = 9; *Cul9*^*−/−*^, *Parkin*^*−/−*^* n* = 10; males: WT *n* = 9; *Cul9*^*−/−*^* n* = 8; *Parkin*^*−/−*^* n* = 10; *Cul9*^*−/−*^, *Parkin*^*−/−*^* n* = 10) (Fig. 4a,b, Supplementary Table S1). We did not observe any significant difference between the wild-type and mutant mice in either sex in this assay. We also examined the olfactory function of the same animals in a buried food test (females: WT *n* = 8; *Cul9*^*−/−*^* n* = 8; *Parkin*^*−/−*^* n* = 8; *Cul9*^*−/−*^, *Parkin*^*−/−*^* n* = 10; males: WT *n* = 9; *Cul9*^*−/−*^* n* = 9; *Parkin*^*−/−*^* n* = 9; *Cul9*^*−/−*^, *Parkin*^*−/−*^* n* = 10) (Fig. 4c,d, Supplementary Table S1). Here too, no consistent difference was detected in any of the single or double knockout mice.

Visual disorders are common among PD patients^44,45^. We therefore tested the visual function of the *Cul9* and *Parkin* single and double KO animals in a Morris water maze where the latency to reach a visible escape platform was measured (females: WT *n* = 10; *Cul9*^*−/−*^* n* = 9; *Parkin*^*−/−*^* n* = 9; *Cul9*^*−/−*^, *Parkin*^*−/−*^* n* = 9; males: WT *n* = 9; *Cul9*^*−/−*^* n* = 8; *Parkin*^*−/−*^* n* = 9; *Cul9*^*−/−*^, *Parkin*^*−/−*^* n* = 9) (Fig. 4e,f, Supplementary Table S1). None of the knockout mutants displayed a reduced ability to find the visible platform compared to the wild-type, and the *Parkin* knockout and *Cul9*, *Parkin* double knockout mice exhibited equivalent performance.

#### Learning and memory in *Cul9*^−/−^, *Parkin*^−/−^ mice

**Figure 5:**
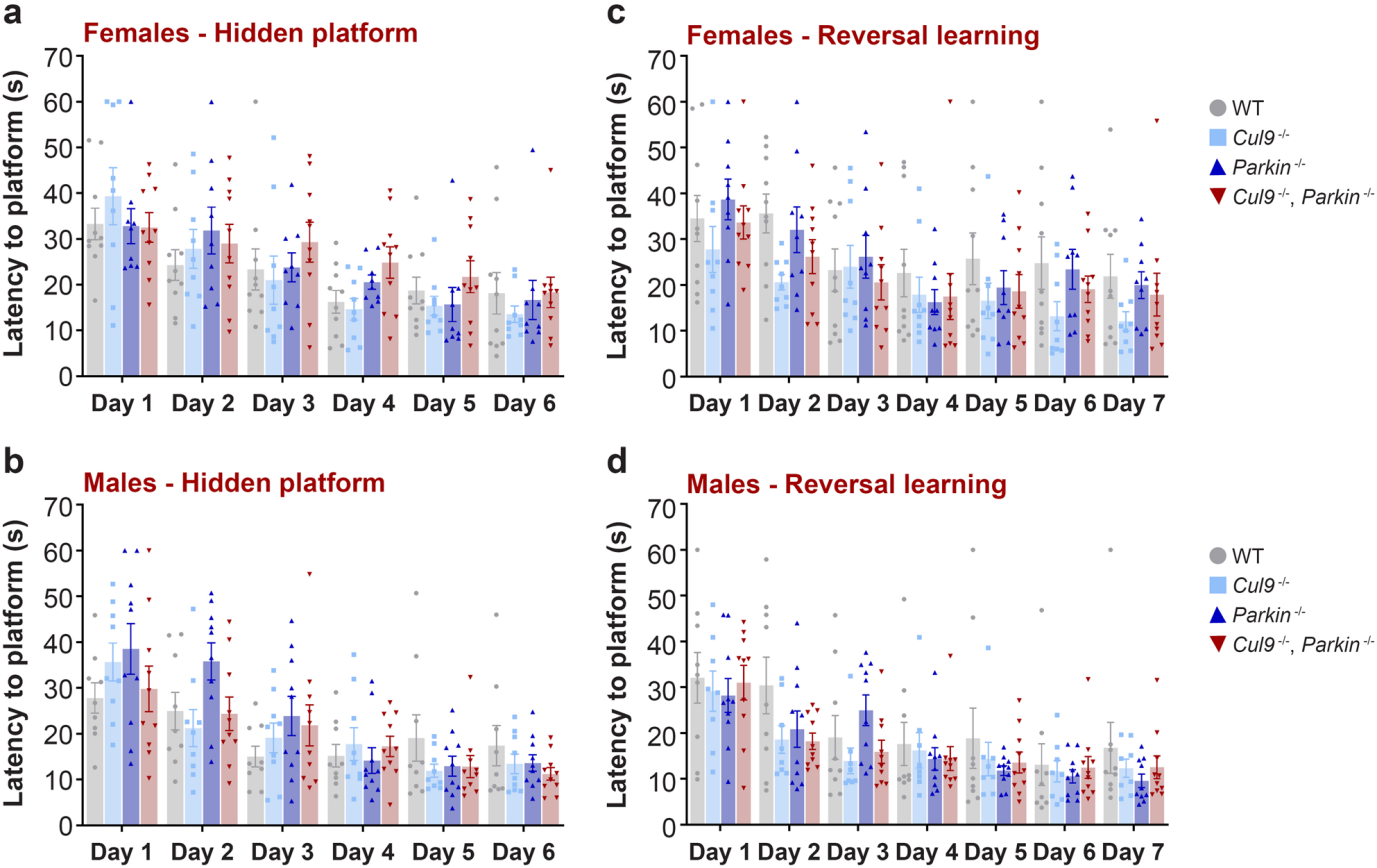
Spatial memory function characterization of *Cul9*^*−/−*^, *Parkin*^*−/−*^ mice. (**a**,**b**) Latency to find a submerged platform in a Morris water maze of *Cul9* and *Parkin* single and double knockout mice (5–7 months old), (females: *n* = 9–10; males: *n* = 9–10). (**c**,**d**) Latency to find the new location of the submerged platform in a Morris water maze of *Cul9* and *Parkin* single and double knockout mice (5–7 months old), (females: *n* = 9–10; males: *n* = 8–10). Means ± SEM are shown, *n* represents the number of independent animals.

Other non-motor deficits reported among PD patients include cognitive dysfunction. For example, attention, working memory and visuospatial function impairment have been reported^4,46^. We therefore subjected the *Cul9* and *Parkin* cohort to a Morris water maze test to measure acquisition and reversal of spatial learning and memory (females: WT *n* = 10; *Cul9*^*−/−*^* n* = 9; *Parkin*^*−/−*^* n* = 9; *Cul9*^*−/−*^, *Parkin*^*−/−*^* n* = 10; males: WT *n* = 9; *Cul9*^*−/−*^* n* = 8; *Parkin*^*−/−*^* n* = 10; *Cul9*^*−/−*^, *Parkin*^*−/−*^* n* = 9). After the initial trial where mice were tested for their ability to find a visible escape platform in an opaque water maze (Fig. 4e,f), mice were then tested for their aptitude to find a submerged escape platform (Fig. 5a,b, Supplementary Table S1). In the last phase of the test, the escape platform was moved to the opposite quadrant of the water maze, and mice were tested for their ability to learn the new location (reversal learning) (Fig. 5c,d, Supplementary Table S1). This phase of the test measured the cognitive flexibility of these mice. Both males and females were able to learn and memorize the location of the hidden platform, and all genotypes exhibited equivalent latency to platform by the end of the trial. Moreover, *Parkin* KO and *Cul9*, *Parkin* double KO animals had comparable performances (Fig. 5a,b). Similarly, in the reversal phase of the learning and memory test, no difference was observed (Fig. 5c,d).

### Loss of *Cul9* and *Parkin* does not affect dopaminergic neurons survival

**Figure 6:**
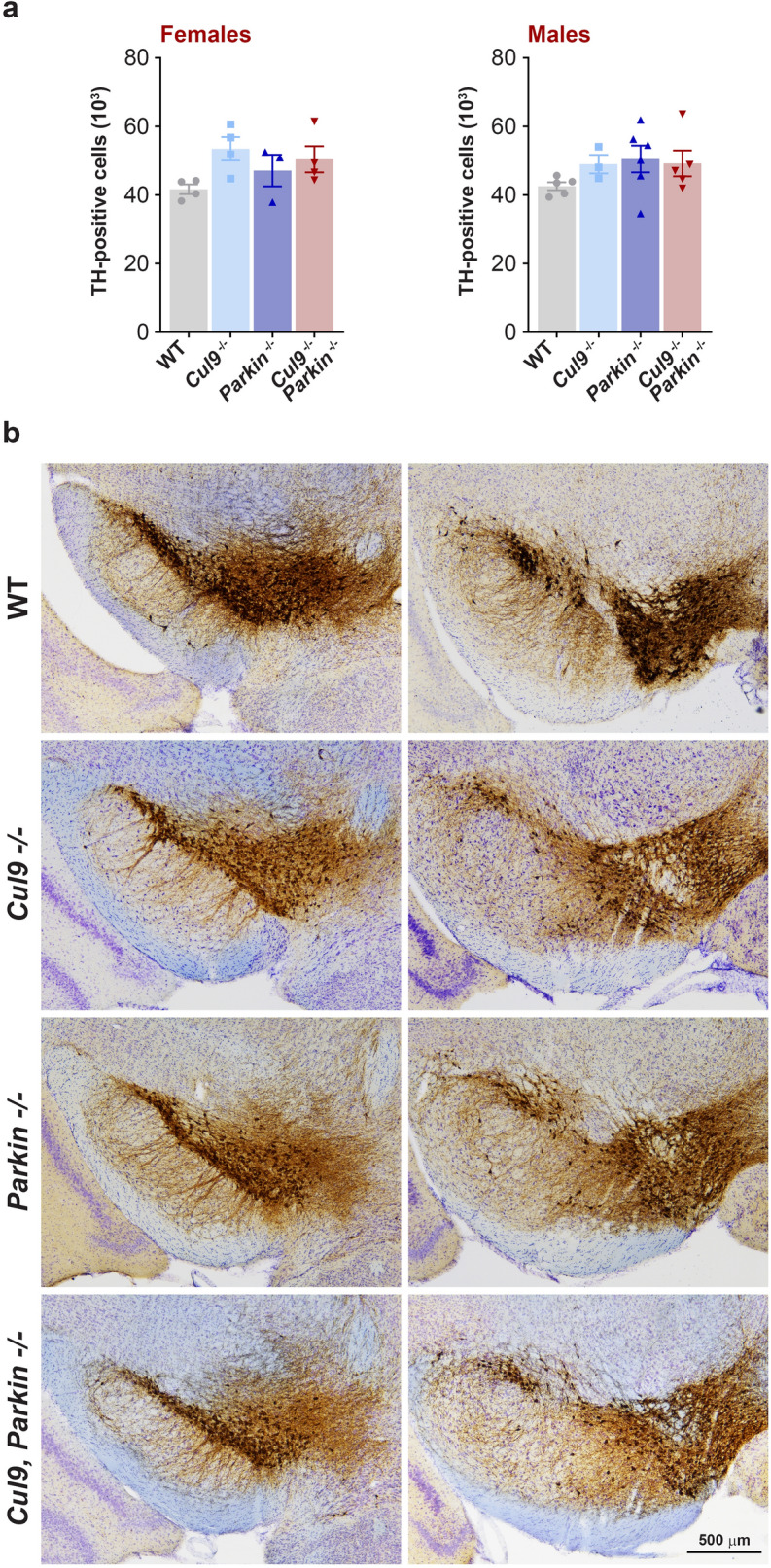
Quantification of dopaminergic neurons in the SNpc of *Cul9*^*−/−*^, *Parkin*^*−/−*^ mice. (**a**) Quantification of tyrosine hydroxylase (TH) expressing neurons in the SNpc of *Cul9* and *Parkin* single and double knockout mice (17–19 months old), (females: *n* = 3–4; males: *n* = 3–6). Means ± SEM are shown, *n* represents the number of independent animals. (**b**) Representative images of coronal brain sections at 2 different levels of the SNpc in mice of the indicated genotype. Nigral dopaminergic neurons were stained for TH (brown) and total neurons were stained with cresyl violet (purple).

A hallmark of PD is the loss of dopaminergic neurons in the substantia nigra. In order to determine if combined loss of *Cul9* and *Parkin* could have an impact on dopaminergic neuron survival, the cohort of mice that underwent the behaviour testing was further aged until they reached 17–19 months. Coronal sections of mouse brains were stained for tyrosine hydroxylase (TH) and the number of dopaminergic neurons in the SNpc was counted (females: WT *n* = 4; *Cul9*^*−/−*^* n* = 4; *Parkin*^*−/−*^* n* = 3; *Cul9*^*−/−*^, *Parkin*^*−/−*^* n* = 4; males: WT *n* = 5; *Cul9*^*−/−*^* n* = 3; *Parkin*^*−/−*^* n* = 6; *Cul9*^*−/−*^, *Parkin*^*−/−*^* n* = 5). None of the mutant mice, male or female, had reduced number of TH-positive neurons compared to wild-type animals (Fig. 6a,b, Supplementary Table S2). In addition, the loss of *Cul9* did not aggravate the effect of *Parkin* loss on dopaminergic neurons viability.

## Discussion

In this study, we conducted a variety of assays in both male and female mice to examine whether *Cul9* deficiency exacerbates the effect of *Parkin* deficiency. Our results show that the combined loss of *Cul9* and *Parkin* does not expose any PD-related pathological features in mice at young or old age.

Our results also extend the finding that the *Parkin*-deficient mice have remarkably no locomotor or behaviour deficits. More broadly, autosomal recessive genetic mouse models have not proven to be robust at replicating the major motor symptoms of PD. Models of *Parkin* deletion alone, in particular, have failed to demonstrate consistent motor defects^26–29,47–51^. For example, discrete deficits such as reduced locomotor activity^28^ and altered gait^26^ have been reported. However, more broadly, the exploratory activity in the open field, the motor balance and coordination in the rotarod and beam tests or the motor strength in the hanging wire and pole tests of the *Parkin* null mice were not abnormal in other studies even at older age (18–24 months)^26–29,47–51^. This suggests that, compared to patients, the effects of *Parkin* loss alone are likely to be more subtle in rodents.

Reports of non-motor behaviour impairments have also been made but with mixed results. For instance, *Parkin*-deficient mice tested for sensorimotor function have been found to have either increased or decreased acoustic startle response^27,29^. In addition, with rare exception^26^, our results for tests of nociceptive function (i.e. hot plate, buried food test) are in agreement with the lack of phenotype of the *Parkin* KO mice reported by others (i.e*.* in adhesive tape removal test, tail-flick test, olfactory discrimination test)^27,49^. Similarly, we and others^27,47^ have not detected any cognitive impairment using the T maze, Morris water maze or the passive avoidance test in *Parkin* mutant mice. However, other studies have detected some degree of memory impairment in the mutant mice using the T and Y mazes^28,49^. With the notable exception of the adult conditional loss of *Parkin* induced by intranigral lentivirus injection^52^, the vast majority of *Parkin*-deficient mutant mice studies also reported an absence of nigrostriatal dopaminergic neurons loss^26–29,47,50^.

Age and gender are the two main risk factors associated with PD. Gender, however, has a complex impact on the aetiology of the disease. Among men, incidence and prevalence of PD are higher, onset is at slightly younger age, and motor symptoms are more severe. Women, on the other hand, seem to have more severe non-motor symptoms including anxiety and depression^53^. Our results, which suggest that the absence of motor deficits in *Parkin*-null mice is not related to the age at which the animals are tested, are consistent with other studies that have followed mice aged up to 18–21 months^26,27,29,47^. Regarding gender, the vast majority of the previous behavioural studies performed on *Parkin* null mice have not examined whether there are differences between males and females. Behaviours have been evaluated for either males only^27,29,47,50,51^, undefined or mixed cohorts^26,28,48,49^. To the best of our knowledge, our study is the first to systematically analyse the behaviour of males and females separately for the *Parkin* KO mice. While overall we did not uncover any sex specific deficits in these mice, we observed that the female but not the male *Parkin* KO had reduced horizontal and vertical locomotor activity as young adult (3–4 months old) but not at an older age (13–14 months old). Most likely due to the inherent variability of the cohort, the differences observed didn’t reach statistical significance and higher number of animals (i.e. higher than 7–9) would be necessary to validate this observation.

A commonly shared explanation for the lack of a PD-like neurodegenerative phenotype in *Parkin* rodents has been the potential redundancy of protective mechanisms in dopaminergic neurons. The premise of our study was that CUL9 could promote neuronal survival despite accumulating mitochondrial damage due to the absence of *Parkin*. Our results, however, do not support the conclusion that the combined deletion of *Cul9* and *Parkin* enhances the susceptibility to PD-related neurodegeneration. One possible explanation for the lack of a phenotype is that mitochondrial defects are rather modest and subtle in *Parkin*-deleted rodent models^23,24,51,54,55^. Indeed, it has been suggested that additional mitophagy pathways could cooperate with the PINK1-Parkin pathway^56^. The E3 ubiquitin ligases SIAH1 and ARIH1 were shown to associate with PINK1 and mediate mitophagy, independently of Parkin, though the ubiquitination of mitochondrial substrates^57,58^. Interestingly, SIAH1 is well known in the context of PD for its ability to ubiquitinate α-synuclein^59^. Other alternative pathways that are independent of PINK1/Parkin involve the mitochondria-associated autophagic receptors BNIP3, BNIP3L/Nix, FUNDC1 or FKBP8 that are able to directly recruit the autophagy machinery through their LIR (LC3-interacting region) domain^60–64^.

Interestingly, exposure to mitochondrial^51,55,65^ or immunogenic stress^66^ can elicit the PD-related pathological behaviour and dopaminergic deficits in *Parkin*-deficient mice. Specifically, accumulation of mitochondrial DNA damage in mouse models has been achieved either by mitochondrial expression of a restriction enzyme (PD-mito-*Pst*1 mice)^51^, mutations in the mitochondrial DNA polymerase POLG (mutator mice)^65^, or in the mitochondrial DNA helicase (Twinkle mice)^55^. These mice, when crossed with the *Parkin*-deficient mice, have all resulted in loss of dopaminergic neurons and motor deficits. It is possible that similar mitochondrial challenges are necessary to reveal a pathological phenotype in *Cul9*-deficient mice.

## Methods

### Reagents

Antibodies for western-blot and immunostaining were obtained from the following suppliers: anti-Parkin (clone PRK8, #sc-32282) and anti-TOM20 (clone F-10, #sc-17764) were from Santa-Cruz Biotechnology, anti-CUL9 (#A300-098A) was from Bethyl Laboratories and anti-β Actin (#A5316) was from Sigma-Aldrich. Anti-mouse Alexa Fluor 647 (#A-31571) and anti-rabbit Alexa Fluor 555 (#A-31572) secondary antibodies for immunofluorescence were obtained from Molecular Probes (Invitrogen). Anti-mouse and anti-rabbit IRDye 680RD (#926-68072) and IRDye 800CW (#926-32213) secondary antibodies for western blotting were obtained from LI-COR. CCCP was obtained from Sigma-Aldrich (#C2759). Plasmid encoding human CUL9 (pcDNA3.1/Zeo (+)) was from Genscript (clone ID OHu15541C) and plasmid encoding YFP-Parkin was a gift from Dr. Richard Youle (Addgene plasmid # 23955).

### Cell culture and transfections

HeLa cells were cultured in RPMI supplemented with 10% foetal calf serum (Sigma) and L-glutamine (Gibco). For plasmid transfections, HeLa cells were seeded at 10^5^ cells per well in 6-well plates, 24 h prior to transfection with Genejet (SignaGen Laboratories, # SL100488). After 24 h, cells were treated with CCCP (10 µM).

### Immunostaining

Cells were grown on coverslips 24 h before transfection and treatment with CCCP. Immunostaining were performed as previously described^67^. Cells were washed with PBS, fixed with 3.5% paraformaldehyde in PBS for 10 min, permeabilized with 0.15% Triton X-100 in PBS for 15 min, and blocked in 2% BSA in PBS for 30 min. Primary antibodies were used at 1:200 for 1 h at room temperature, followed by secondary antibodies at 1:1,000 for 1 h at room temperature. Final washing included incubation with 500 nM Hoechst (Sigma-Aldrich) for 10 min. Cells were mounted with Slow Fade (Molecular Probes). Images were acquired by Olympus FluoView 3000RS confocal microscope with 20x/0.75 objective in the UNC Neuroscience Microscopy Core facility using Olympus FluoView (FV31S-SW) software. For each individual experiment, quantification of Parkin translocation was estimated by counting a minimum of 3 × 100 cells in 3 fields of view for each treatment.

### Western blot analysis

Following CCCP treatment, whole-cell lysates were prepared with 100 µl of Laemmli buffer. Samples were boiled for 10 min, ran on 10% or 8% SDS PAGE gels, and transferred onto Immobilon-FL PVDF membranes (Millipore). After blocking (TBS, 1% ovalbumin, 1% fish gelatin, 0.05% tween 20), membranes were probed with primary antibodies (1:1000) and infrared dye-conjugated secondary antibodies (1:10,000) and imaged using an Odyssey CLx imager (LI-COR).

### Mice

All animal handling and experiments were carried out in accordance with the National Institutes of Health Guide for Care and Use of Laboratory Animals and as approved by the Animal Care and Use Committee of the University of North Carolina (UNC). Mice were housed 2–5 per cage in a 12 h light, 12 h dark cycle. Food and water were provided ad libitum. Mice for which the exon 3 of the *Parkin* gene was replaced in-frame by the coding sequence for EGFP^26^ were obtained from The Jackson Laboratory (JAX stock #006582, mixed C57BL/6 × 129 genetic background). Mice deleted for exons 2–7 of the *Cul9* gene^68^ (C57BL/6 genetic background) were a generous gift of Dr. Yue Xiong (University of North Carolina at Chapel Hill, Chapel Hill, NC, USA).

### RT-qPCR analysis

cDNA libraries were prepared using iScript cDNA Synthesis Kit (Biorad, #1708891) from total RNA (1 µg) extracted from mouse brain and heart using DirectZol RNA kit (Zymo Research, #R2051). The following primers were used: *mGapdh* forward: TGTGTCCGTCGTGGATCTGA; *mGapdh* reverse: CCTGCTTCACCACCTTCTTGA; *mCul9* forward: TGGCACATGCTAGAGATCCTG; *mCul9* reverse: GGCTGGAGATACGGCAGTG; *mParkin* forward: CGTGTGATTTTTGCCGGGAAG; *mParkin* reverse: GGTCCACTCGTGTCAAGCTC. Real-time PCR reactions were performed with Power Up SYBR green master mix (Applied Biosystems, #A25742) on a QuantStudio 3 thermocycler (Applied Biosystems). Relative quantification was estimated using the ΔΔCt method. Each mRNA relative level was normalized to *Gapdh*.

### Mouse behavioural analysis

Mouse behaviour was tested as previously described^69^. Specifically, the following assays were performed:

#### Open field test

Exploratory activity in a novel environment was assessed by a 60 min trial in an open field chamber (41 cm × 41 cm × 30 cm) crossed by a grid of photobeams (VersaMax system, AccuScan Instruments). Counts were taken of the number of photobeams broken during the trial in 5 min intervals, with separate measures for total distance travelled and fine movements (the repeated breaking of the same set of photobeams).

#### Wire-hang test for grip strength

At the start of the test, subjects were placed on a large metal cage lid. The lid was gently shaken to induce the mouse to grip onto the metal grid. The cage top was then flipped over, and latency for the mouse to fall from the lid was recorded during the 60 s test.

#### Rotarod test

Subjects were tested for motor coordination and learning on an accelerating rotarod (Ugo Basile, Stoelting Co.). On a first test session, mice were given three trials, with 45 s between each trial. An additional test was given 48 h later. The initial speed was set at 3 rpm, with a progressive increase to a maximum of 30 rpm across a total 5 min trial. Measures were taken for latency to fall from the top of the rotating barrel.

#### Hot-plate test

To investigate thermal or pain sensitivity animals were placed in a tall plastic cylinder on a hot plate (IITC) heated to 55 °C, and examined for any reaction (hind paw licking, jumping or vocalization) to the heated surface. The animals were immediately removed after any reaction to the hot plate, with a maximum test length of 30 s. Measures were taken of latency to respond.

#### Test for olfactory function

Three days before the test, an unfamiliar food (Froot Loops, Kellogg’s Co.) was placed overnight in the home cages of the subject mice. Observations of consumption were made to ensure that the novel food was palatable to the animals. The day before the test, all food was removed from the cages. After 16–20 h of food deprivation, each mouse was placed in a large, clean tub cage containing 3 cm deep paper chip bedding and allowed to explore for 5 min. The mice were then removed from the cage, and one piece of the food reward was buried in the cage bedding. The animal was returned to the cage and given 15 min to locate the buried food. Measures were taken of latency to find the food.

#### Acquisition and reversal learning in the Morris water maze

The water maze consists of a large circular pool (diameter = 122 cm) filled with water made opaque with nontoxic white paint (45 cm deep, 24–26 °C), located in a room with numerous visual cues. To test for their ability to find a visible escape platform, mice were given 4 trials per day, across 2 days. For each trial, mice were placed at one of four possible locations (randomly ordered), and then given 60 s to find the visible platform. Measures of latency to find the platform were obtained via an automated tracking system (Noldus Ethovision). To evaluate acquisition of spatial learning, mice were tested for their ability to find a submerged escape platform (diameter = 12 cm). Mice were given 4 trials per day, as described above, with a maximum of 9 days of testing. Criterion for learning was a group average of 15 s to find the escape platform. After reaching criterion in the acquisition phase, mice were further tested for reversal learning where the escape platform was moved to the opposite quadrant of the water maze. Mice were tested for their ability to learn the new location of the hidden platform.

### Tissue collection and stereological estimate of SNpc DA neurons

Mice were anesthetized using isoflurane and transcardially perfused with PBS followed by 4% paraformaldehyde. Brains were post-fixed in 4% paraformaldehyde overnight before cryoprotection in 30% (w/v) sucrose solution in PBS. Coronal brain sections (40 µm) were prepared on a freezing microtome and stored in 0.1% sodium azide and PBS at 4 °C. Tissue was immunohistochemically stained for tyrosine hydroxylase (Millipore AB152, 1:1000) and counterstained with 0.1% cresyl violet (Nissl staining) as previously described^70^.

Counting of dopamine nigral neurons (TH-positive) and total nigral neurons (Nissl-positive) was conducted using an Optical Fractionator probe (MicroBrightField) as previous described^70^. Briefly, the SNpc was outlined under low magnification (4 ×) on a Nikon eclipse 90i microscope from tissue spaced 240 µm apart. Neurons were counted using a 40 × objective in a counting frame of 50 µm × 50 µm with a grid size of 90 µm × 120 µm and a dissector height of 15 µm with 1 µm guard zones. All counts were conducted by an investigator blinded to genotype. Nigral images were acquired using a Leica DMi8 inverted microscope.

### Statistical analysis

Data are expressed as mean ± SEM. For all experiments, *n* represents the number of independent animals used. Sample sizes were estimated based on similar samples sizes reported in the literature. All behaviour and stereology analyses were performed blinded to the genotype. Biochemical analyses were performed without blinding. GraphPad Prism v.8.3.1 was used for all data visualization and statistical analysis. Outliers were identified using the ROUT method with a Q value of 5%. The Student’s t-test (unpaired, two-tailed) with Welch's correction was used for analysis of two groups with normal distribution. For all assays comparing multiple groups with normal data distributions, a one-way Analysis of Variance (ANOVA) with a post-hoc Tukey's multiple comparisons test was performed. For assays where samples did not present a normal distribution, a Kruskal–Wallis test with a post-hoc Dunn's multiple comparisons test was used. The Log-rank (Mantel-Cox) test was performed for survival analysis. For behavioral assays based on repeated measurements, data with normal distribution were analyzed using two-way repeated measures ANOVA with a 95% CI followed by a post-hoc Tukey's multiple comparisons test. A p-value lower than 0.05 was considered statistically significant.

## Supplementary information

Supplementary Information.
