# Supplementary material for: Characterization of a Cul9–Parkin double knockout mouse model for Parkinson’s disease
Source: Sci Rep. 2020 Oct 9;10:16886. doi: 10.1038/s41598-020-73854-y (PMC7547682; doi:10.1038/s41598-020-73854-y)
Supplement: Supplementary file 1 — Supplementary Information. [file 41598_2020_73854_MOESM1_ESM.pdf]

Supplementary Information

Characterization of a *Cul9* – *Parkin* double knockout mouse model for Parkinson's disease

Emilie Hollville, Valerie Joers, Ayumi Nakamura, Vijay Swahari, Malú G. Tansey, Sheryl S. Moy and Mohanish Deshmukh

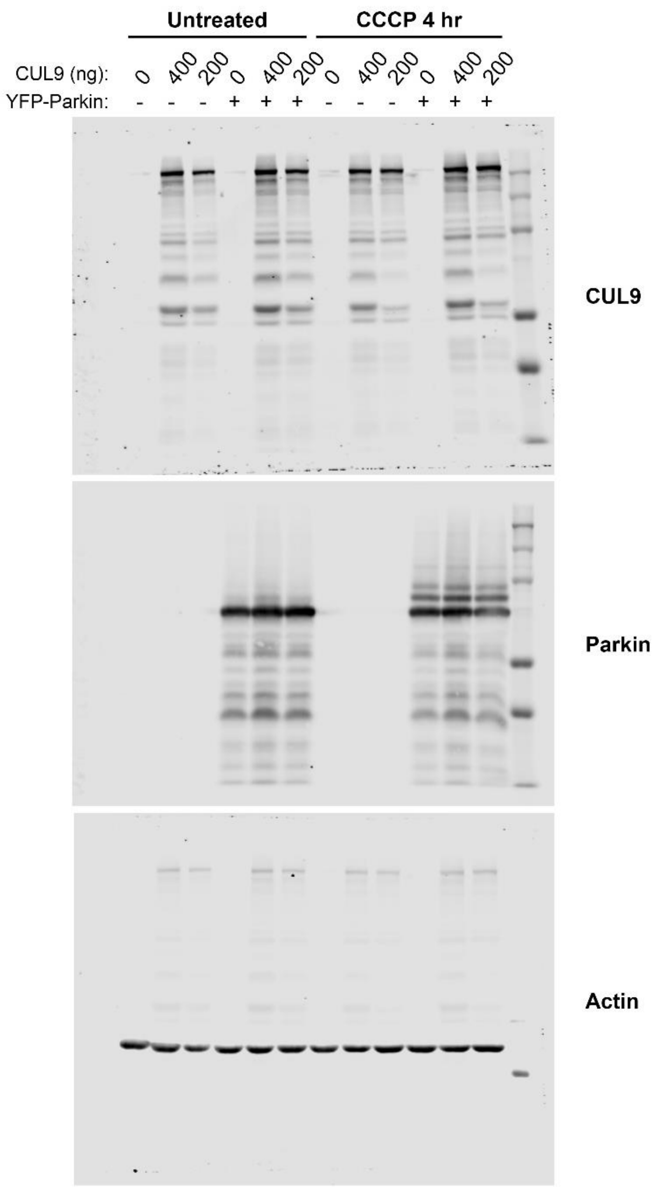

Supplementary Figure S1. Overexpression of CUL9 and Parkin in HeLa cells. HeLa cells were transfected with the indicated amount of plasmid encoding CUL9 along with YFP-Parkin construct (200 ng). Cells were treated with CCCP (10 µM) for 4 hr and whole-cell lysates were analysed by immunoblotting. Full-length immunoblots of blots presented in Fig. 1c.

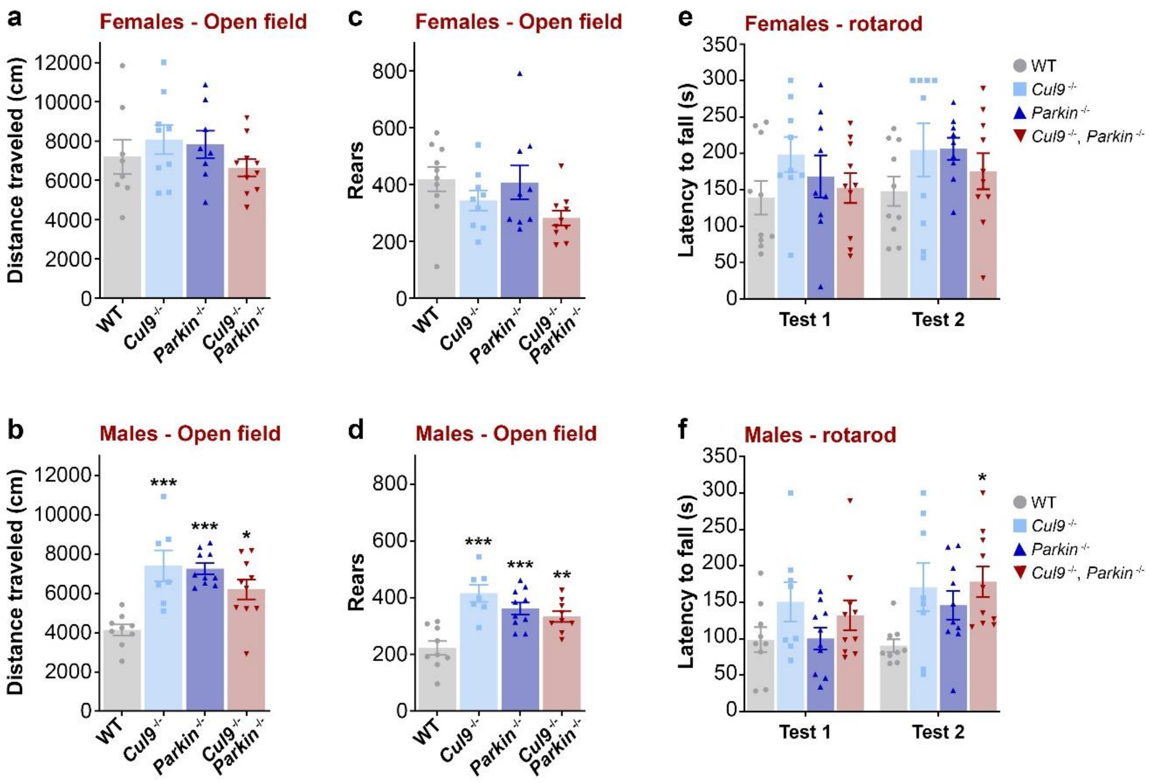

Supplementary Figure S2. Locomotor function characterization of aged *Cul9*$^{-/-}$*, Parkin*$^{-/-}$ mice. (**a, b**) Total distance travelled and (**c, d**) total number of rears in the open field (1 hr) of *Cul9* and *Parkin* single and double knockout mice (13-14 months old), (females distance: *n* = 8-10; males distance: *n* = 7-10; females rears: *n* = 9-10; males rears: *n* = 7-10). One-way ANOVA with post-hoc Tukey's multiple comparisons test (males distance: WT vs. *Cul9*$^{-/-}$, ***p = 0.0003; WT vs. *Parkin*$^{-/-}$, ***p = 0.0002; WT vs. *Cul9*$^{-/-}$*, Parkin*$^{-/-}$, *p = 0.0141; males rearing: WT vs. *Cul9*$^{-/-}$, ***p < 0.0001; WT vs. *Parkin*$^{-/-}$, ***p = 0.0008; WT vs. *Cul9*$^{-/-}$*, Parkin*$^{-/-}$, **p = 0.0100). (**e, f**) Latency to fall from an accelerating rotarod (5 minute test, 48 hr between test 1 and test 2) of *Cul9* and *Parkin* single and double knockout mice (11-12 months old), (females: *n* = 9-10; males: *n* = 8-10). Two-way repeated measures ANOVA with post-hoc Tukey's multiple comparisons test (males test 2: WT vs. *Cul9*$^{-/-}$*, Parkin*$^{-/-}$, *p = 0.0173). Means ± SEM are shown, *n* represents the number of independent animals.

		Females				Males			
	age (months)	Wild-type (n)	*Cul9* KO (n)	*Parkin* KO (n)	*Cul9, Parkin* KO (n)	Wild-type (n)	*Cul9* KO (n)	*Parkin* KO (n)	*Cul9, Parkin* KO (n)
Locomotor function									
Distance travelled (cm)	3-4	7687 ± 573 (7)	9723 ± 939 (9)	5911 ± 504 (9)	5913 ± 392 (10)	5751 ± 494 (9)	6030 ± 105 (7)	7225 ± 469 (10)	5688 ± 404 (10)
Vertical activity (rears)	3-4	356 ± 39 (10)	303 ± 38 (9)	245 ± 27 (9)	213 ± 27 (10)	317 ± 77 (9)	313 ± 30 (9)	388 ± 35 (10)	263 ± 20 (10)
Rotarod test 2, latency to fall (s)	4-5	217.6 ± 21.8 (10)	246.0 ± 19.2 (9)	269.0 ± 10.6 (9)	264.1 ± 16.1 (9)	153.3 ± 16.5 (9)	261.6.3 ± 19.4 (9)	260.6 ± 23.0 (10)	262.2 ± 16.4 (10)
Distance travelled (cm)	13-14	7202 ± 876 (8)	8085 ± 753 (9)	7840 ± 696 (8)	6655 ± 444 (10)	4139 ± 275 (9)	7403 ± 789 (7)	7261 ± 273 (10)	6205 ± 509 (10)
Vertical activity (rears)	13-14	420 ± 43 (10)	344 ± 35 (9)	408 ± 60 (9)	283 ± 26 (10)	223 ± 25 (9)	416 ± 31 (7)	362 ± 21 (10)	334 ± 19 (9)
Rotarod test 2, latency to fall (s)	11-12	148.2 ± 20.3 (10)	204.9 ± 36.7 (9)	206.3 ± 15.2 (9)	175.5 ± 24.7 (10)	90.4 ± 8.7 (9)	170.8 ± 33.1 (8)	145.9 ± 19.6 (10)	178.3 ± 20.9 (10)
Sensory function									
Hot plate, latency (s)	6-7	27.0 ± 1.0 (10)	30.0 ± 0.1 (9)	29.0 ± 0.8 (9)	27.0 ± 1.0 (10)	28.0 ± 1.0 (9)	29.0 ± 0.6 (8)	28.0 ± 1.0 (10)	28.0 ± 0.8 (10)
Burried food, latency (s)	4-5	47 ± 11 (8)	122 ± 57 (8)	80 ± 23 (8)	109 ± 20 (10)	128 ± 46 (9)	144 ± 30 (9)	107 ± 32 (9)	62 ± 9 (10)
Visible platform day 1, latency (s)	4-5	21.3 ± 2.8 (10)	20.6 ± 2.3 (9)	24.1 ± 2.3 (9)	22.9 ± 3.3 (9)	23.1 ± 3.7 (9)	18.6 ± 3.1 (8)	17.5 ± 2.8 (9)	18.5 ± 2.4 (9)
Cognitive function									
Spatial learning day 6, latency (s)	5-7	18.1 ± 2.5 (10)	13.6 ± 1.8 (9)	16.7 ± 4.3 (9)	18.3 ± 3.3 (10)	17.4 ± 4.4 (9)	13.4 ± 2.1 (9)	13.6 ± 1.8 (10)	11.2 ± 1.4 (10)
Reversal learning day 7, latency (s)	5-7	21.9 ± 4.8 (10)	12.1 ± 2.1 (9)	20.0 ± 2.9 (9)	17.9 ± 4.6 (10)	16.8 ± 5.5 (9)	12.3 ± 2.0 (8)	9.6 ± 1.5 (10)	12.5 ± 2.5 (10)

Supplementary Table S1. Summary of behavioural tests results for *Cul9* and *Parkin* single and double knockout mice.

		Females				Males			
	age (months)	Wild-type (n)	*Cul9* KO (n)	*Parkin* KO (n)	*Cul9, Parkin* KO (n)	Wild-type (n)	*Cul9* KO (n)	*Parkin* KO (n)	*Cul9, Parkin* KO (n)
Stereology									
TH-positive cells	17-19	4170 ± 143 (4)	5355 ± 342 (4)	4717 ± 464 (3)	5046 ± 381 (4)	4261 ± 116 (5)	4904 ± 270 (3)	5056 ± 391 (6)	4926 ± 378 (5)
Nissl-positive cells	17-19	6692 ± 1017 (4)	8607 ± 976 (4)	8028 ± 289 (3)	8433 ± 538 (4)	6771 ± 818 (5)	9513 ± 951 (3)	8718 ± 848 (6)	7445 ± 941 (5)

Supplementary Table S2. Stereological analysis of the SNpc of *Cul9* and *Parkin* single and double knockout mice.
